# Interaction of background Ca^2+^ influx, sarcoplasmic reticulum threshold and heart failure in determining propensity for Ca^2+^ waves in sheep heart

**DOI:** 10.1113/JP282168

**Published:** 2022-03-20

**Authors:** David C. Hutchings, George W. P. Madders, Barbara C. Niort, Elizabeth F. Bode, Caitlin A. Waddell, Lori S. Woods, Katharine M. Dibb, David A. Eisner, Andrew W. Trafford

**Affiliations:** ^1^ Unit of Cardiac Physiology Division of Cardiovascular Sciences Faculty of Biology Medicine and Health Manchester Academic Health Science Centre University of Manchester Manchester UK; ^2^ Manchester University NHS Foundation Trust Manchester UK

**Keywords:** Ca^2+^, heart failure, sarcoplasmic reticulum, threshold, waves

## Abstract

**Abstract:**

Ventricular arrhythmias can cause death in heart failure (HF). A trigger is the occurrence of Ca^2+^ waves which activate a Na^+^‐Ca^2+^ exchange (NCX) current, leading to delayed after‐depolarisations and triggered action potentials. Waves arise when sarcoplasmic reticulum (SR) Ca^2+^ content reaches a threshold and are commonly induced experimentally by raising external Ca^2+^, although the mechanism by which this causes waves is unclear and was the focus of this study. Intracellular Ca^2+^ was measured in voltage‐clamped ventricular myocytes from both control sheep and those subjected to rapid pacing to produce HF. Threshold SR Ca^2+^ content was determined by applying caffeine (10  mM) following a wave and integrating wave and caffeine‐induced NCX currents. Raising external Ca^2+^ induced waves in a greater proportion of HF cells than control. The associated increase of SR Ca^2+^ content was smaller in HF due to a lower threshold. Raising external Ca^2+^ had no effect on total influx via the L‐type Ca^2+^ current, *I*_Ca‐L_, and increased efflux on NCX. Analysis of sarcolemmal fluxes revealed substantial background Ca^2+^ entry which sustains Ca^2+^ efflux during waves in the steady state. Wave frequency and background Ca^2+^ entry were decreased by Gd^3+^ or the TRPC6 inhibitor BI 749327. These agents also blocked Mn^2+^ entry. Inhibiting connexin hemi‐channels, TRPC1/4/5, L‐type channels or NCX had no effect on background entry. In conclusion, raising external Ca^2+^ induces waves via a background Ca^2+^ influx through TRPC6 channels. The greater propensity to waves in HF results from increased background entry and decreased threshold SR content.

**Key points:**

- Heart failure is a pro‐arrhythmic state and arrhythmias are a major cause of death.
- At the cellular level, Ca^2+^ waves resulting in delayed after‐depolarisations are a key trigger of arrhythmias. Ca^2+^ waves arise when the sarcoplasmic reticulum (SR) becomes overloaded with Ca^2+^.
- We investigate the mechanism by which raising external Ca^2+^ causes waves, and how this is modified in heart failure.
- We demonstrate that a novel sarcolemmal background Ca^2+^ influx via the TRPC6 channel is responsible for SR Ca^2+^ overload and Ca^2+^ waves.
- The increased propensity for Ca^2+^ waves in heart failure results from an increase of background influx, and a lower threshold SR content.
- The results of the present study highlight a novel mechanism by which Ca^2+^ waves may arise in heart failure, providing a basis for future work and novel therapeutic targets.

## Graphical abstract

**Abstract figure legend** Raising external Ca^2+^ (1) leads to a background Ca^2+^ influx via TRPC6 channels (2). This Ca^2+^ is pumped into the sarcoplasmic reticulum via SERCA leading to a rise in SR Ca^2+^ content (3). When SR Ca^2+^ content reaches a threshold, spontaneous Ca^2+^ release leads to propagating Ca^2+^ waves (4). In heart failure, the background Ca^2+^ influx is increased and SR threshold decreased, resulting in a greater propensity to Ca^2+^ waves.

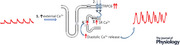

## Introduction

Cardiac contraction is activated by an increase of cytoplasmic Ca^2+^ concentration ([Ca^2+^]_i_). The bulk of this Ca^2+^ is provided by release from the sarcoplasmic reticulum (SR) by a mechanism known as calcium induced calcium release (CICR) in which Ca^2+^ entering the cell, via the L‐type Ca^2+^ current, produces a local increase of [Ca^2+^]_i_ which opens the SR Ca^2+^ release channel (ryanodine receptor, RyR). See Bers (2008) and Eisner *et al*. (2017) for reviews. It is well known that SR Ca^2+^ release can also occur in the absence of triggering L‐type Ca^2+^ current leading to abnormal waves of CICR (Wier *et al*. 1987). These waves activate delayed afterdepolarizations and thence ventricular ectopic beats and arrhythmias (Ferrier *et al*. 1973; Rosen *et al*. 1973; Lederer & Tsien, 1976). Ca^2+^ waves and their arrhythmogenic consequences occur more frequently in heart failure (Pogwizd *et al*. 2001). Ca^2+^ waves are initiated when the SR Ca^2+^ content exceeds a threshold level and this can occur in one of two ways. (1) If the threshold is decreased, as occurs when the RyR open probability is increased by mutations, for example in catecholaminergic polymorphic ventricular tachycardia (CPVT) (Jiang *et al*. 2005; Kashimura *et al*. 2010). A decreased threshold may account for the increased propensity for waves in heart failure (Belevych *et al*. 2007; Maxwell *et al*. 2012). (2) Waves and delayed afterdepolarizations can also occur when the myocyte is overloaded with Ca^2+^such that SR Ca^2+^ content is increased above the threshold level (Díaz *et al*. 1997; Jiang *et al*. 2004) as was first demonstrated for digitalis intoxication (Ferrier *et al*. 1973; for review see Venetucci *et al*. 2008).

A commonly used experimental tool to produce Ca^2+^overload is to elevate the extracellular Ca^2+^ concentration (Kass *et al*. 1978; Hayashi *et al*. 1994; Cheng *et al*. 1996; Díaz *et al*. 1997; Minamikawa *et al*. 1997; Lukyanenko *et al*. 1999; Yang *et al*. 2007; Wasserstrom *et al*. 2010). It is, however, unclear by what mechanism elevating extracellular Ca^2+^ increases SR Ca^2+^ content to the threshold for waves to develop. One possibility might be increased influx through the L‐type Ca^2+^ current. However, the effects of an increase of L‐type current on SR content are complicated; loading of the SR by increased influx is opposed by increased release and the net effect is hard to predict (Trafford *et al*. 2001). Another explanation is a decrease of Ca^2+^ efflux on sodium calcium exchange (NCX) due to the increased driving force against which it must transport. Finally, as recently reviewed (Eisner *et al*. 2020), there are other, as yet inadequately characterized, mechanisms by which Ca^2+^ can enter the cell (Terracciano & MacLeod, 1996; Kupittayanant *et al*. 2006; Hutchings *et al*. 2021), including Trp channels (Camacho Londono *et al*. 2015), and connexin hemi channels (Wang *et al*. 2012; De Smet *et al*. 2021).

The aim of the work in this paper was to characterize the mechanisms by which elevating extracellular Ca^2+^ concentration increases the occurrence of Ca^2+^ waves in sheep ventricular myocytes taken from both control animals and in heart failure. We find that this is associated with elevated SR Ca^2+^ content but this is not a consequence of either increased L‐type Ca^2+^ current or decreased NCX but, rather, of background Ca^2+^ entry. The majority of this background entry appears to be via TRPC6 channels. Our findings indicate that arrhythmogenic Ca^2+^ waves are produced more easily in myocytes from heart failure animals due to a combination of a larger background influx and a lower threshold SR Ca^2+^ content.

## Methods

### Ethical approval

All procedures involving the use of animals were performed in accordance with The United Kingdom Animals (Scientific Procedures) Act, 1986 and European Union Directive 2010/63. Institutional approval was obtained from The University of Manchester Animal Welfare and Ethical Review Board. Furthermore, the study accords with the ARRIVE guidelines (Percie du Sert *et al*. 2020).

### Induction of heart failure

Female Welsh mountain sheep were group‐housed, at 19−21°C, in a 12:12 h light:dark cycle. Animals had *ad libitum* access to drinking water, and were fed hay and ruminant concentrate. No animals were excluded from the study. Heart failure was induced in 13 adult animals (∼18 months age, weight 31.9 ± 3.7 kg) via rapid pacing as previously described (Dibb *et al*. 2009; Briston *et al*. 2011; Lawless *et al*. 2019). Briefly, under general anaesthesia (isoflurane 1−4%) animals underwent transvenous insertion of a pacing lead with active fixation to the apex of the right ventricle, and connected to a pacemaker buried subcutaneously in the right pre‐scapular position. Subcutaneous Meloxicam (0.5 mg kg^−1^) was administered for perioperative analgesia, and Enrofloxacin (5 mg kg^−1^) or oxytetracycline (20 mg kg^−1^) administered for perioperative antibiosis. Following a recovery period (at least 7 days) rapid pacing was commenced (210 beats per minute; bpm). Animals were monitored on a daily basis for features of heart failure (cough, dyspnoea). Heart failure animals developed symptoms at 51 ± 16 days, at which point they were humanely killed for isolation of cells by anaesthetic overdose (200 mg kg^−1^ intravenous pentobarbitone). Heparin (10,000–25,000 i.u.) was used to prevent coagulation.

### Cellular studies

Left ventricular myocytes were isolated from sheep using a collagenase and protease digestion technique as described previously (Dibb *et al*. 2004; Briston *et al*. 2011).

Voltage clamp was imposed using the whole cell technique. Following rupture of the patch, access resistance (∼5 MΩ) was overcome using the switch clamp facility of the Axoclamp‐2B voltage clamp amplifier (Axon Instruments, Union City, CA, USA). Electrodes (2–3 MΩ resistance) were filled with a pipette solution containing (in mM): CsCl, 118; MgCl_2,_ 4.0; CaCl_2_, 0.28; sodium phosphocreatine, 3; HEPES, 10; CsEGTA, 0.02; Na_2_ATP, 3.1; Na_2_GTP, 0.42; pH 7.2 with CsOH. For all experiments under voltage clamp, intracellular Ca^2+^ concentration ([Ca^2+^]_i_) was measured using the indicator Fura‐2 (pentapotassium salt; 100  μM, Invitrogen), loaded via the patch pipette. As indicated in the figure legends, fluorescence was excited either at wavelengths of 365 and 380 nm or 340 and 380 nm and emitted fluorescence detected at 510 ± 10 nm. After subtracting background fluorescence, the ratio of light excited at 340 or 365 nm to that excited at 380 nm was used to measure changes in [Ca^2+^]_i_.

Cells were held at a holding potential of −40 mV and depolarizing pulses to 10 mV applied at 0.5 Hz; L‐type Ca^2+^ current and NCX currents were measured as previously described (Trafford *et al*. 1997). Cells were superfused with (in mM): NaCl, 140; KCl, 4.0; MgCl_2_, 1; HEPES, 10; glucose, 10; CaCl_2_, 1.8; probenecid, 2; 4‐aminopyridine, 5; BaCl_2_, 0.1; pH 7.34 with NaOH. Ca^2+^ waves were induced by increasing external Ca^2+^ to 10 mM. In some experiments (Fig. 2) it was necessary to elevate external Ca^2+^ to 15 mM to produce waves.

SR content was measured at −40 mV by rapidly applying 10 mM caffeine (Sigma‐Aldrich, UK) to discharge Ca^2+^ from the SR, and integrating the resulting inward NCX current (*I*_NCX_) (Varro *et al*. 1993). To determine threshold SR Ca^2+^ content to induce waves, caffeine (10 mM) was added immediately following a wave. The sum of the integrals of the wave and caffeine‐induced NCX currents was taken as threshold (Kashimura *et al*. 2010). For all experiments in 1.8 mM external Ca^2+^, total efflux was estimated by multiplying *I*_NCX_ efflux by a correction factor (1.44) to account for Ca^2+^ removal by PMCA. For experiments in 10 mM external Ca^2+^, no correction factor was used as PMCA removal is inhibited under these conditions (Bassani *et al*. 1992).

In separate experiments, pharmacological inhibitors were used to examine the identity of the background Ca^2+^ influx in unpatched spontaneously waving cells (Figs 6*Ab* and 7). [Ca^2+^]_i_ was measured using the acetoxymethyl ester (AM) form of Fura‐4F (Life Technologies, USA). Fluorescence excited at wavelengths of 340 and 380 nm. 18β‐Glycyrrhetinic acid (100 μM, Sigma, UK) was used to inhibit connnexin hemichannels (Guan *et al*. 1996; Vaiyapuri *et al*. 2012), nicardipine (5 μM, Stratech, UK) for inhibition of L‐type Ca^2+^ channels (Sun *et al*. 1999), BI 749327 (100 nM, MedChem Express, USA) for inhibition of TRPC6 channels (Lin *et al*. 2019), and Pico145 (13 nM, Generon, UK) for inhibition of TRPC1/4/5 channels (Rubaiy *et al*. 2017). Gadolinium chloride (100 μM, Bio‐Techne Ltd, UK) was used as a non‐specific inhibitor of background influx (Kupittayanant *et al*. 2006). Inhibitors were dissolved in DMSO (final concentration not exceeding 0.1% v/v) with the exception of gadolinium (dissolved in water). For each experiment, inhibitors were paired with a vehicle control of the same volume.

Finally, to further investigate the background influx, manganese (Mn^2+^) quench was performed, as previously described (Camacho Londono *et al*. 2015). Experiments were performed in Ca^2+^‐free superfusion solution. Myocytes were AM‐loaded with Fura‐4 and excited at near‐isosbestic wavelength (365 nm). Initial control recordings showed a slow decline in the *F*_365_ signal related to a combination of photobleaching and indicator loss. MnCl_2_ (1 mM, Sigma, UK) was then rapidly applied, leading to Mn^2+^ entry via background Ca^2+^ channels and quenching of the Fura signal. The rate by which Mn^2+^ quenches Fura provides a measure of the rate of Mn^2+^ entry via background channels. Quench rates were determined after subtracting the rate of photobleaching/indicator loss. The rate of quench was normalized to that from in a cell from the same animal in the absence of inhibitors. The effect of the inhibitors 18β‐glycyrrhetinic acid, Pico145, BI 749327, and gadolinium on the quench rate were determined. In some experiments, the rate of decline in the presence of inhibitors was slower than the prior control rate, resulting in apparent negative quench rate. This is probably because of the control rate being exponential rather than linear. In such cases the rate was assigned a value of zero for calculations

All cellular experiments were performed at 37°C.

### Statistics

Data are presented as means ± standard deviation for *n* cells/*N* animals. As in previous work (Caldwell *et al*. 2014), when comparisons were made between control and HF animals and multiple cells studied from the same animal, linear mixed modelling (SPSS Statistics, IBM, USA) was performed thus accounting for the nested (clustered) design of the experiment (Eisner, 2021). Data was log10 transformed before linear mixed modelling to achieve a normal distribution (Keene, 1995). Categorical variables were compared between groups using the Fischer's exact or chi‐squared tests as appropriate. For Mn^2+^ quench experiments, the rate of quench in the presence of a putative inhibitor was paired with that in the absence of inhibitor in a cell from the same isolation using a Wilcoxon matched‐pairs signed rank test. Exact *P* values are stated if *P* > 0.0001.

## Results

### Effects of elevation of external Ca^2+^ concentration on Ca^2+^ cycling

**Figure 1:**
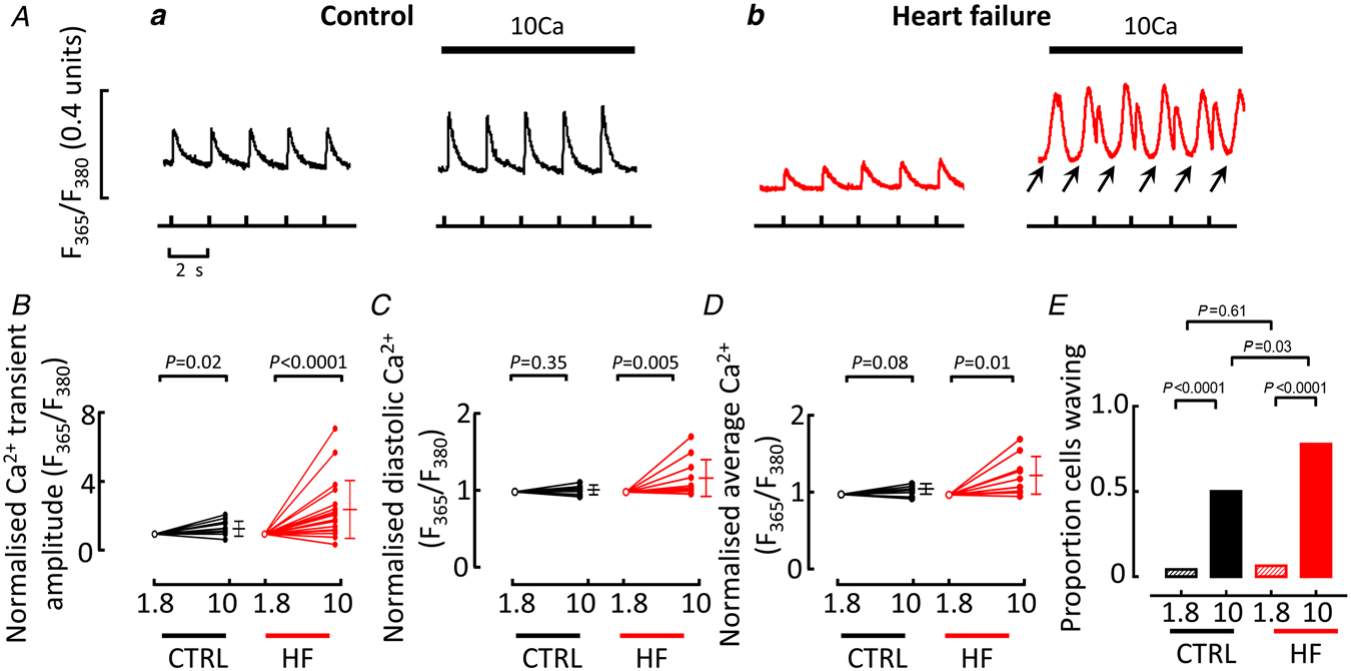
Effects of increasing external Ca^2+^ concentration in control and heart failure cells *A*, specimen records showing effects of elevating Ca^2+^ from 1.8 to 10 mM in a control (*a*) and heart failure (HF) (*b*) myocytes. In this and subsequent figures, cells were stimulated with 100 ms duration pulses from a holding potential of −40 to 10 mV, applied at 0.5 Hz. Arrows indicate Ca^2+^ waves. *B*–*D*, summary data (normalized to 1.8 mM Ca^2+^) showing the effects of increasing external Ca^2+^ on Ca^2+^ transient amplitude (*B*), diastolic [Ca^2+^]_i_ (*C*) and average [Ca^2+^]_i_ (*D*). *E*, summary data of the proportion of cells showing waves. Mean ± SD shown to the right of data in 10 mM Ca^2+^. For Ca^2+^ transient amplitude; control 11 cells/8 animals one sample *t* test, HF 19 cells/8 animals Wilcoxon matched pairs signed rank test. For diastolic [Ca^2+^]_i_; control 9 cells/6 animals one sample *t* test, HF 12 cells/7 animals Wilcoxon matched pairs signed rank test. For average Ca^2+^; control 8 cells/5 animals, HF 11 cells/7 animals, one sample *t* test for both comparisons. For proportion of cells waving; control 1.8 mM Ca^2+^ 117 cells/41 animals, control 10 mM Ca^2+^ 30 cells/16 animals, HF 1.8 mM Ca^2+^ 31 cells/10 animals, HF 10 mM Ca^2+^ 27 cells/10 animals, chi‐squared test for all comparisons. [Colour figure can be viewed at wileyonlinelibrary.com]

Previous work has shown that, in this sheep tachypacing model, heart failure decreases the amplitude of the systolic Ca^2+^ transient (Briston *et al*. 2011; Lawless *et al*. 2019). Figure 1*A* shows that increasing external Ca^2+^ concentration from 1.8 to 10 mM increased the amplitude in both control (*a*) and heart failure (*b*) cells, although the percentage increase was greater in heart failure (141 ± 166%; mean ± SD) than control (37 ± 43%; mean ± SD) (Fig. 1*B*). In heart failure (but not control) cells, raising external Ca^2+^ increased diastolic (*C*) and average (*D*) [Ca^2+^]_i_. Figure 1*E* shows that in control cells in 1.8 mM Ca^2+,^ only a small proportion of cells showed Ca^2+^ waves and this fraction increased in 10 mM Ca^2+^ to 50%. The propensity to wave was greater in heart failure (78%, *P* = 0.03). Subsequent experiments were designed to investigate the role of SR Ca^2+^ content in this increased incidence of waves in elevated external Ca^2+^.

### Effects of external Ca^2+^ concentration on sarcoplasmic reticulum Ca^2+^ content

**Figure 2:**
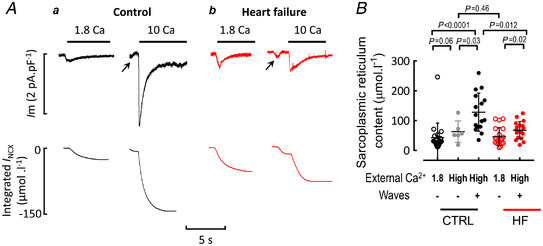
Effects of external Ca^2+^concentration on SR Ca^2+^ content and threshold for waves *A*, original data. Traces show: top, membrane current; bottom, integral of current. Records are taken from representative examples from control (*a*) and HF (*b*) myocytes. In both, the left‐hand traces were recorded in 1.8 mM Ca^2+^ and the right‐hand in 10 mM. 10 mM caffeine was applied for the period shown by the horizontal bars. Arrows show inward currents produced by Ca^2+^ waves. *B*, summary data. In this, and subsequent diagrams, error bars denote ± SD for both control and heart failure, the left‐hand points (open symbols) show SR Ca^2+^ content measured in 1.8 mM Ca^2+^ (in the absence of waves: 21 cells from 15 animals in control and 20 cells from 7 animals in HF). The right‐hand points (+waves) show the SR Ca^2+^ content in those cells which displayed waves in elevated Ca^2+^. This was achieved in 9 control cells (from 7 animals) and 18 HF cells (from 9 animals) by elevating external Ca^2+^ to 10 mM, and in 7 control cells (from 4 animals) by elevating external Ca^2+^ to 15 mM. Cells from control animals which did not display waves in high Ca^2+^ are also shown (grey symbols, marked ‘−waves’, total 6 cells from 5 animals; two of which were in 15 mM Ca^2+^ and 4 in 10 mM Ca^2+^). For control 1.8 Ca *vs*. control high Ca ‘−waves’, Mann‐Whitney test. For control 1.8 Ca *vs*. control high Ca ‘+waves’, Mann‐Whitney test. For control high Ca ‘+waves’ *vs*. control high Ca ‘−waves’, unpaired *t* test. For control high Ca ‘+waves’ *vs*. HF high Ca ‘+waves’, mixed effects linear mixed modelling. For HF 1.8 Ca *vs*. HF high Ca, Mann‐Whitney test. [Colour figure can be viewed at wileyonlinelibrary.com]

In the experiments illustrated in Fig. 2*A*, the SR Ca^2+^ content was measured from the integral of the caffeine‐evoked NCX current. Waves were absent in 1.8 mM external Ca^2+^ in both the control (*a*) and heart failure cells (*b*), but appeared in 10 mM. This was accompanied by an increase of SR Ca^2+^ content as shown by the integral of the caffeine‐evoked NCX current. The summary data of Fig. 2*B* (black points) show the measurements for all those control cells which were induced to wave by elevation of external Ca^2+^ concentration. The appearance of waves was associated with an increase of SR Ca^2+^ content from 42 ± 48 to 128 ± 61 μmol l^−1^ (mean ± SD). Figure 2*Ab* and the mean data of Fig. 2*B* demonstrate that, in heart failure, the induction of waves by elevation of external Ca^2+^ was associated with a smaller increase of SR Ca^2+^ than was the case for control cells; SR Ca^2+^ content increased from 45 ± 29 to 67 ± 28 μmol l^−1^ (mean ± SD). The lower SR Ca^2+^ content in heart failure indicates that the threshold for Ca^2+^ waves is lower in heart failure cells than control. This explains why SR Ca^2+^ content rises less in heart failure as it is limited by the production of Ca^2+^ waves. To further illustrate this, control cells which were below threshold and not displaying waves in high Ca^2+^ are shown as grey points in the summary data. These cells had lower SR contents than control cells with waves, but similar SR contents to HF cells with waves.

The difference of threshold for production of Ca^2+^ waves provides one explanation as to why heart failure cells are more likely to exhibit Ca^2+^ waves. We have, however, argued previously that a difference of threshold by itself is insufficient to produce waves (Venetucci *et al*. 2007). Specifically, something must maintain the SR Ca^2+^ content to balance the extra efflux resulting from Ca^2+^ waves. Subsequent experiments were therefore designed to investigate the change of Ca^2+^ fluxes.

### Effects on the L‐type Ca^2+^ current

**Figure 3:**
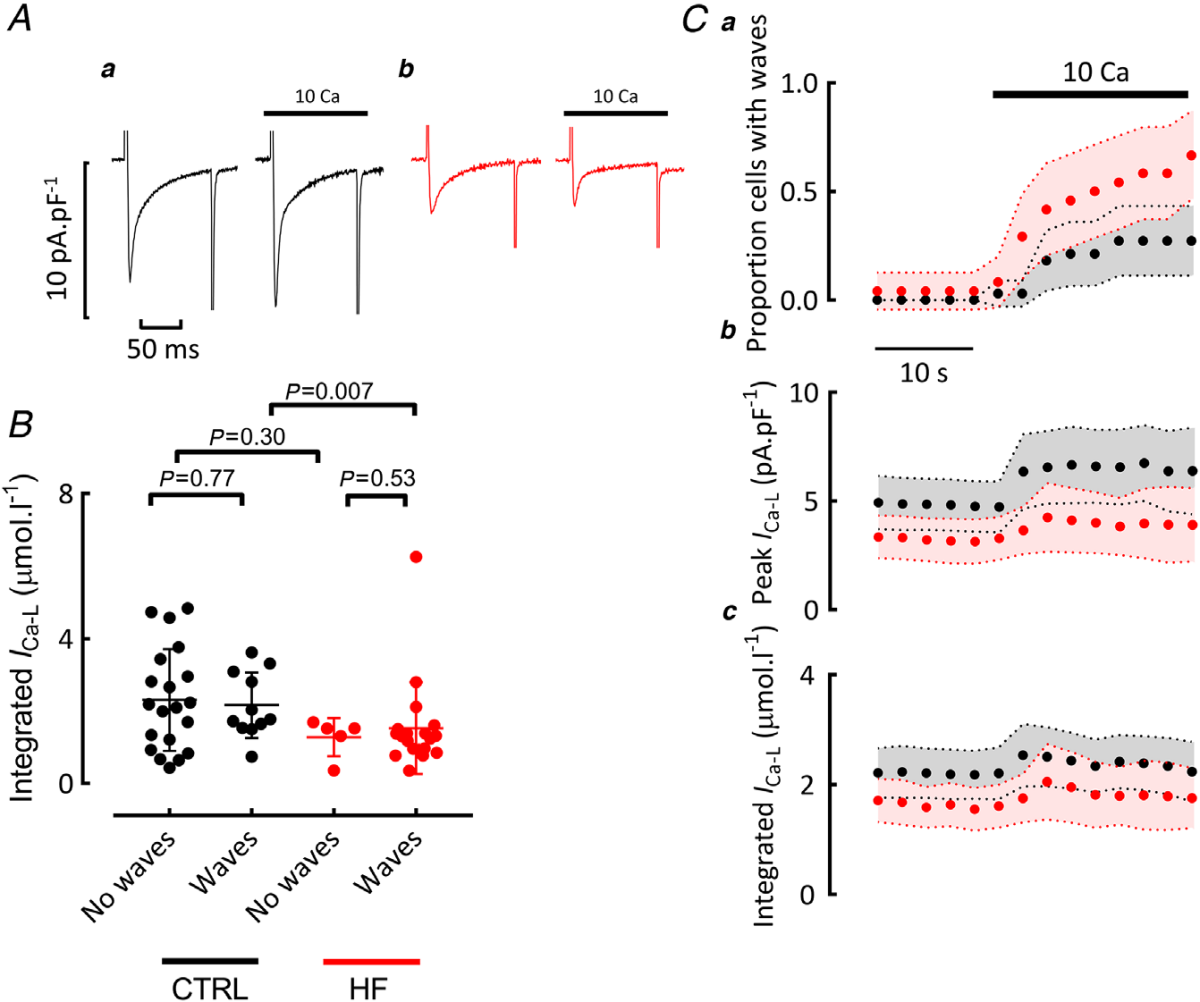
The effects of external Ca^2+^ concentration on the L‐type Ca^2+^ current *A*, specimen paired records showing the effects of elevating external Ca^2+^ from 1.8 to 10 mM. In all panels 100 ms duration depolarizing pulses were applied at 0.5 Hz to +10 mV from a holding potential of −40 mV. Panels show: *a*, control; *b*, heart failure. In both panels the left‐hand trace was obtained in 1.8 and the right‐hand in 10 mM external Ca^2+^ from the same cell. *B*, integral of the L‐type Ca^2+^ current in 10 mM external Ca^2+^. Black symbols from control cells, red heart failure. In both, data are separated by whether the cells showed waves or not. Control: no waves 20 cells/14 animals, with waves 11 cells/5 animals. Heart failure: no waves 5 cells/3 animals, with waves 19 cells/8 animals. For control no waves *vs*. with waves, unpaired *t* test. For HF no waves *vs*. with waves, Mann‐Whitney test. For control no waves *vs*. HF no waves, mixed effects linear mixed modelling. For control with waves *vs*. HF with waves, Mann‐Whitney test. *C*, time course of mean data (31 control and 24 heart failure cells). Graphs show: *a*, fraction of cells displaying waves; *b*, mean peak L‐type Ca^2+^ current; *c*, mean integral of L‐type current. Black symbols, control; red symbols, heart failure. External Ca^2+^ concentration was increased from 1.8 to 10 mM for the period shown. Shaded areas show 95% confidence limits. [Colour figure can be viewed at wileyonlinelibrary.com]

Figure 3 addresses the question as to whether the increases of SR content and wave probability produced by elevating external Ca^2+^ involve changes in the L‐type Ca^2+^ current. In control cells, elevating Ca^2+^ increases the peak L‐type current (Fig. 3 *Aa* and *Cb*). On average, in 10 mM Ca^2+^ in the steady state^,^ the amplitude of the L‐type current increased from 4.57 ± 3.33 to 6.58 ± 4.59 pA·pF^−1^ (mean ± SD, *P < *0.0001) but this was not accompanied by any change of total Ca^2+^ entry, assessed from the integral (Fig. 3*B* and *Cc*), as the current inactivates more quickly. In heart failure cells, the increase of wave probability was not associated with changes of either amplitude or integral of the L‐type Ca^2+^ current. In 10 mM Ca^2+^, the integral of the L‐type current was lower in heart failure than in control (*P* = 0.03). Thus, influx via the L‐type current does not determine whether cells wave in either cell type, and therefore cannot explain the greater propensity to waves in heart failure.

### Effects on Ca^2+^ efflux and background influx

**Figure 4:**
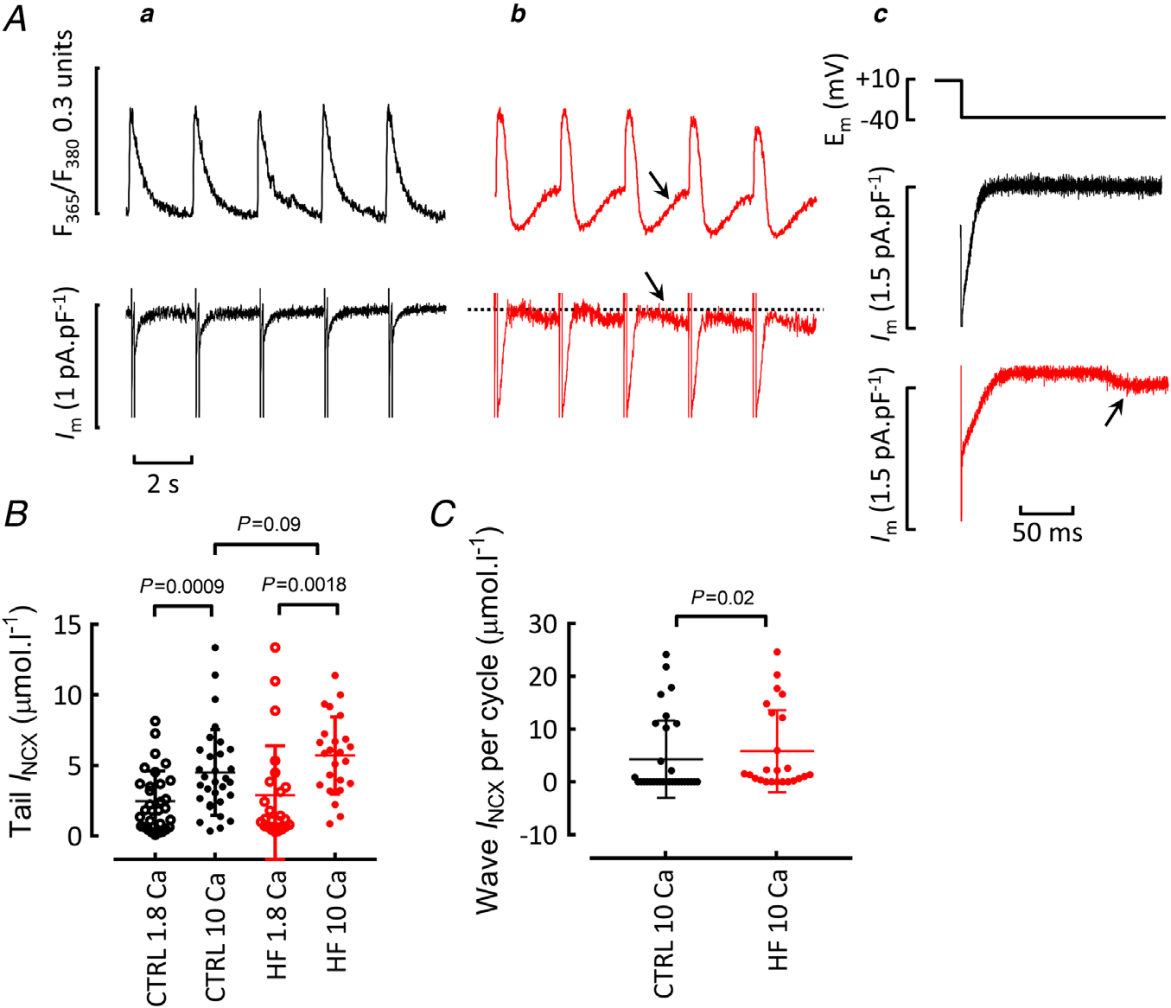
Measurement of Ca^2+^ efflux in elevated external Ca^2+^ *A*, original records: top, [Ca^2+^]_i_, bottom, membrane current. *a*, control; *b*, heart failure; *c*, expanded, averaged (five sweeps) membrane current records. All data obtained in 10 mM external Ca^2+^. Arrows denote a Ca^2+^ wave and accompanying inward current. *B*, average Ca^2+^ efflux on NCX during the Ca^2+^ transient (tail). Data shown from both 1.8 and 10 mM external Ca^2+^ in control and HF. Control: 31 cells/18 animals, HF 24 cells/9 animals. For comparisons between 1.8 and 10 mM Ca^2+^ (in both HF and control), paired *t* tests. For comparison between HF and control, mixed effects linear mixed modelling. *C*, average Ca^2+^ efflux on NCX per cycle during waves. Control: 31 cells/18 animals, HF 24 cells/9 animals, Mann‐Whitney test. [Colour figure can be viewed at wileyonlinelibrary.com]

**Figure 5:**
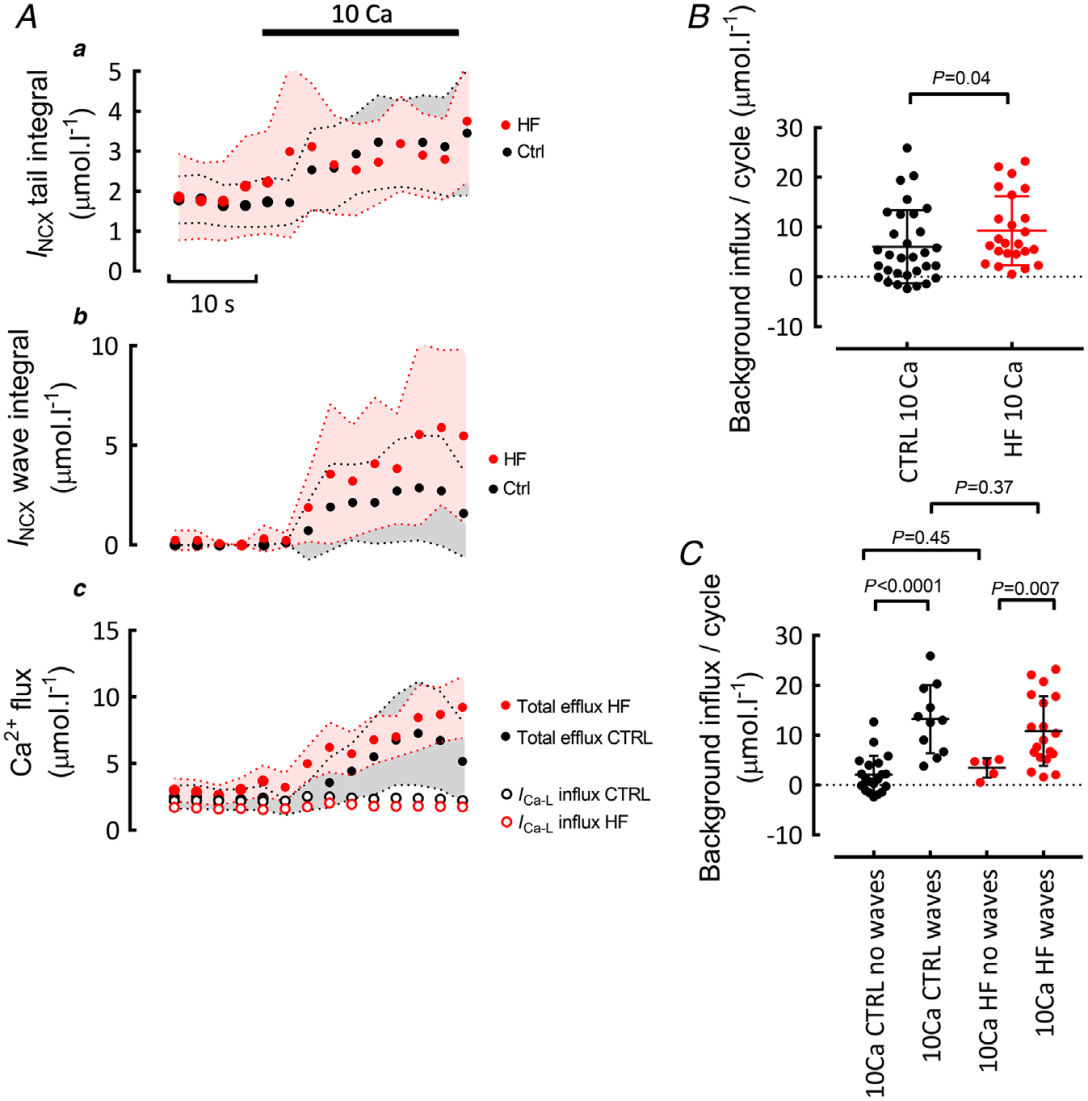
Estimation of background flux *A*, time course of mean data showing the effects of elevating external Ca^2+^ from 1.8 to 10 mM: *a*, efflux during Ca^2+^ transient (tail); *b*, efflux on waves; *c*, total efflux (filled symbols) compared with Ca^2+^ influx on L‐type current (open symbols). Shaded areas show 95% confidence limits. *B*, background influx in the steady state in 10 mM external Ca^2+^ in control (left) and heart failure (right). Control 31 cells/18 animals, HF 24 cells/9 animals, mixed effects linear mixed modelling. *C*, background influx as a function of both whether waves are present and cell type. Control: no waves 20 cells/14 animals, with waves 11 cells/5 animals. HF: no waves 5 cells/4 animals, with waves 19 cells/9 animals. For control, no waves *vs*. with waves, Mann‐Whitney test. For HF, no waves *vs*. with waves, Mann‐Whitney test. For comparisons between HF and control, mixed effects linear mixed modelling. [Colour figure can be viewed at wileyonlinelibrary.com]

Figure 4 shows steady state measurements of Ca^2+^ efflux recorded from cells exposed to 10 mM external Ca^2+^. In cells without Ca^2+^ waves, such as the control myocyte illustrated in Fig. 4 *Aa*, the NCX current was observed as a ‘tail’ during the decay of [Ca^2+^]_i_ on repolarization. As shown in Fig. 4*B*, in both control and heart failure, increasing external Ca^2+^ increased the NCX tail efflux to similar levels. Therefore, decreased NCX activity cannot be the explanation of the increase of SR Ca^2+^ load. When waves were present (Fig. 4*Ab* and *c*), the NCX tail current was followed by an NCX current activated by the Ca^2+^ wave. Mean data for the wave‐associated Ca^2+^ efflux are shown in Fig. 4*C*. There is a considerable spread of values, with those at the zero level representing cells which did not have waves. The average wave‐associated efflux is greater in heart failure than control because a greater fraction of cells displays Ca^2+^ waves. The time course of changes of Ca^2+^ efflux produced by elevating external Ca^2+^ is shown in Fig. 5*A*. Elevation of external Ca^2+^ increases Ca^2+^ efflux via NCX on both the tail (*a*) and waves (*b*). Figure 5*Ac* (filled symbols) plots the sum of these two components of Ca^2+^ efflux. In 1.8 mM this efflux is equal to the influx on the L‐type current (open symbols) but greatly exceeds it in 10 mM. In control cells, elevation of external Ca^2+^ increases Ca^2+^ efflux per cycle (2 s) from 2.5 ± 2.1 to 8.2 ± 7.1 μmol l^−1^ (mean ± SD) while, in heart failure, the respective values are 2.9 ± 3.5 and 10.7 ± 7.3 μmol l^−1^ (both comparisons between the last data points in 1.8 and 10 mM Ca^2+^). In the steady state, total Ca^2+^ efflux must equal influx so the fact that influx through the L‐type channel is smaller than the efflux means that there must be another ‘background’ component of influx. The magnitude of this influx is shown in Fig. 5*B*; on average the background influx is greater in heart failure (9.3 ± 6.8 μmol l^−1^ per cycle; mean ± SD) than in control (6.0 ± 7.2). The analysis of Fig. 5*C* measures the background influx as a function, not only of cell type but, in addition, whether the cells are showing Ca^2+^ waves or not. Analysis of those cells without waves in 10 mM Ca^2+^ shows no significant difference in background influx between control and heart failure. In both control and heart failure, those cells that show waves have a greater background influx than those that do not and, finally, the background influx is similar in control (13.2 ± 6.5 μmol l^−1^ per cycle; mean ± SD) and heart failure (10.8 ± 6.8) cells that wave. A background Ca^2+^ influx has been demonstrated previously but its identity was unknown (Kupittayanant *et al*. 2006). The remainder of the experiments were therefore designed to characterize this flux.

### Identity of the background influx

**Figure 6:**
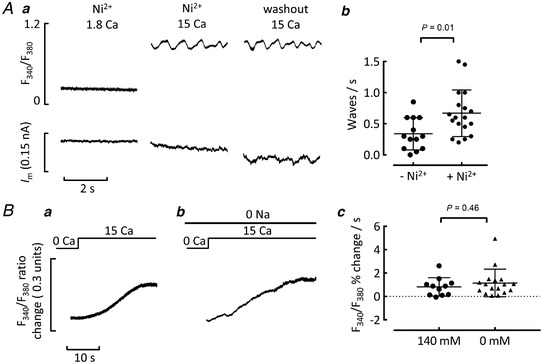
Effect of NCX block on Ca^2+^waves and background Ca^2+^ entry *Aa*, representative recordings from a single cell under voltage clamp. Panels show (from left to right): Ni^2+^ (10 mM) and 1.8 mM Ca^2+^; Ni^2+^ and 15 mM Ca^2+^; Ni^2+^ washout in 15 mM Ca^2+^. *Ab*, summary data for effects of Ni^2+^ (10 mM) on waves in unpatched cells in 10 mM Ca^2+^. Unpaired data. *B*, representative increases in [Ca^2+^]_i_ when cells were exposed to 15 mM external Ca^2+^. The cells had been in Ca^2+^‐free solution for at least 2 min before raising Ca^2+^, and caffeine (10 mM) was present throughout to prevent Ca^2+^ uptake into the SR. Records show: *a*, control; *b*, Na‐free (a different cell). *c*, summary data for the maximum rate of rise. For *Ab*, *n* = 13 cells/4 animals Ctrl *vs. n* = 18 cells/3 animals Ni^2+^, unpaired *t* test. For *Bc*, *n* = 11 cells/3 animals in 140 mM Na^+^, *n* = 17 cells/3 animals in 0 mM Na^+^, Mann‐Whitney test.

We first examined whether Ca^2+^ could be entering between waves by NCX acting in reverse. Ni^2+^ was used to inhibit NCX (Kimura *et al*. 1987). Initial experiments under voltage clamp confirmed Ni^2+^ (10 mM) blocked *I*_NCX_ in 15 mM Ca (reversible loss of wave *I*_NCX_ current in three cells exposed to Ni^2+^, Fig. 6*Aa*). Ni^2+^ also blocked *I*_Ca‐L_. Further experiments were performed in unpatched cells pre‐exposed to Ni^2+^ for at least 30 s. Raising external Ca^2+^ to 15 mM in the presence of Ni^2+^ induced waves in a similar proportion of cells to control (waving in Ni^2+^ 52.0% *vs*. Ctrl 59.7%, *P = *0.51). Ni^2+^ increased the frequency of waves (Fig. 6*Ab*). In 12 cells which did not wave in Ni^2+^, washing Ni^2+^ out did not induce waves in any cells (not shown).

Figure 6*B* shows an alternative method of assessing the contribution of NCX to the background influx. The record in Fig. 6*Ba* shows the typical rise in [Ca^2+^]_i_ when 15 mM Ca^2+^ solution was applied to a cell which had been pre‐exposed to a Ca^2+^‐free solution. To block NCX completely, the cell in Fig. 6*Bb* was pre‐exposed to Ca^2+^ and Na^+^‐free solution (Na^+^ replaced by Li^+^); here a similar rise in [Ca^2+^]_i_ is seen, indicating the background Ca^2+^ entry is not via reverse‐mode NCX (see also summary data Fig. 6*Bc*).

**Figure 7:**
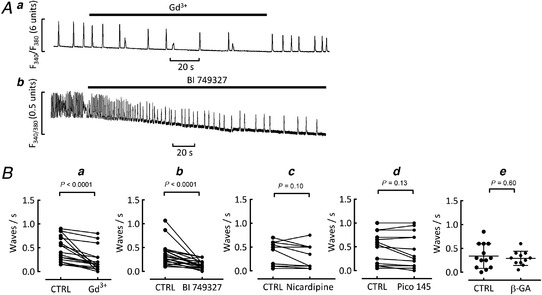
Effect of inhibitors on spontaneous Ca^2+^ waves Waves were induced in control cells by raising external Ca^2+^ to 15 mM. *A*, representative Ca^2+^ recordings in waving cells exposed to Gd^3+^ and its washout (*a*) and BI 749327 (*b*). *B*, mean effects of inhibitors on wave frequency: *a*, mean effect of Gd, paired data from *n* = 18 cells/3 animals. Wilcoxon matched‐pairs signed rank test; *b*, mean effect of BI 749327, Wilcoxon matched‐pairs signed rank test on paired data from *n* = 20 cells/4 animals; *c*, mean effect of nicardipine, paired *t* test from *n* = 11 cells/3 animals; *d*, mean effect of Pico145 on wave frequency, paired *t* test from *n* = 13 cells/3 animals; *e*, mean effect of pre‐incubation with β‐glycrrhetinic acid on wave frequency, unpaired *t* test from *n* = 13 cells/4 animals (control) and 11 cells/3 animals (β‐GA).

To investigate other possible candidates for the background influx, specific inhibitors were tested in cells displaying spontaneous waves in high Ca^2+^ (15 mM; Fig. 7). Under these conditions, a decrease of background influx should decrease wave frequency. Gadolinium (Gd^3+^, 100 μM) and the TRPC6 inhibitor, BI 749327 (100 nM) both reduced the frequency of waves (Fig. 7*Aa* and *b*, and *Ba* and *b*). In contrast, block of *I*_Ca‐L_ with nicardipine (5 μM) had no effect on wave frequency (Fig. 7*Bc*). Neither the application of the TRPC1/4/5 channel inhibitor Pico145 nor pre‐incubating cells with β‐glycrrhetinic acid had any effect on waves (Fig. 7*Bd* and *e*).

**Figure 8:**
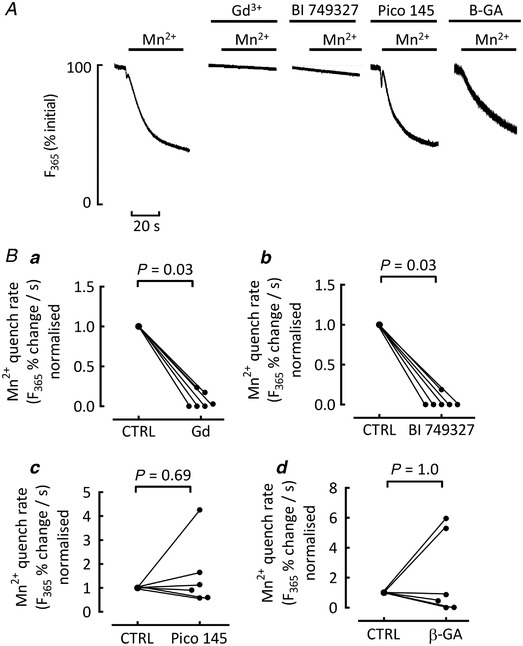
Assessment of background influx with Mn^2+^ quench *A*, representative recordings of Fura signal quench (*F*_365_) in single cells exposed to Mn^2+^ (1 mM). The effects of Mn^2+^ were tested in control cells (left), and when exposed to gadolinium, BI 749327, Pico 145 and β‐glycrrhetinic acid. *B*, summary mean data. For analysis, cells were randomly paired with control cells from the same animal and the rate of quench normalized to the control value. For each inhibitor *Ba–d*, *n* = 6 cells/3 animals, Wilcoxon ranked pairs signed rank test.

Subsequent experiments were designed to measure background Ca^2+^ influx more directly using the quench of Ca^2+^ ‐sensitive indicators produced by Mn^2+^ (Camacho Londono *et al*. 2015). Figure 8*A* shows typical quenches of the Fura‐2 signal when Mn^2+^ was applied. The quench was suppressed in the presence of Gd^3+^ and BI 749327; see also Fig. 8*Ba* and *b*. In contrast, exposing cells to Pico145 (to inhibit TRPC 1/4/5 channels) or β‐glycrrhetinic acid (to inhibit connexin hemichannels) had no effect on Mn^2+^ quench rates (Fig. 8 *Bc* and *d*).

The above findings suggest the background Ca^2+^ entry responsible for Ca^2+^ waves is independent of *I*_Ca‐L_ and *I*_NCX_, is Gd sensitive, and appears to be carried via TRPC6 channels, with no apparent role for TRPC1/4/5 channels or connexin hemichannels.

## Discussion

The main results of this paper are that, in elevated external Ca^2+^, ventricular myocytes from sheep with heart failure are more likely to show Ca^2+^ waves than are those from control animals. Two factors are responsible for this: (i) lower SR Ca^2+^ threshold for waves in heart failure; and (ii) a larger proportion of HF cells have a high background Ca^2+^ influx. Finally, we have shown that Ca^2+^ entry through TRPC6 channels is the most likely candidate for the molecular nature of this background Ca^2+^ entry.

### SR Ca^2+^ threshold

We find that the SR Ca^2+^ threshold at which waves occur in heart failure is about half of the value observed in control cells, a result which is qualitatively in agreement with previous work (Belevych *et al*. 2007; Maxwell *et al*. 2012) and may result from RyR dysfunction and an increase of RyR open probability, as a consequence of factors such as phosphorylation (Marx *et al*. 2000; Ai *et al*. 2005; van Oort *et al*. 2010), oxidation (Terentyev *et al*. 2008), and decreased S‐nitrosylation (Gonzalez *et al*. 2007). This lower threshold and the consequent Ca^2+^ release from the SR during waves explain why elevation of external Ca^2+^ concentration increases SR Ca^2+^ content less in heart failure cells than in control. It is worth noting that whilst SR Ca^2+^ content is the same in control and heart failure in 1.8 mM Ca^2+^, the lower threshold SR Ca^2+^ content in heart failure means that the normal SR Ca^2+^ content is closer to threshold, and thus one potential explanation for the greater propensity for Ca^2+^ waves. While the decrease of threshold in heart failure is important, it cannot be the only explanation for the greater occurrence of waves in heart failure. This is because, in the steady state, the Ca^2+^ efflux on waves must be balanced by additional Ca^2+^ influx (Venetucci *et al*. 2007; Eisner *et al*. 2013). It is therefore important to consider Ca^2+^ influx and, more generally, Ca^2+^ flux balance.

### Ca^2+^ flux balance

#### L‐type Ca^2+^ channel and NCX

In control cells we find that an increase of external Ca^2+^ concentration produced a small increase of the amplitude of the L‐type current. It is unclear why no similar effect was seen in heart failure. In neither control nor heart failure, however, was there any effect on the amount of Ca^2+^ entering through this current as assessed from its integral. It appears that the increase of amplitude is balanced by faster inactivation. This lack of effect on Ca^2+^ entry via the L‐type current contrasts with the marked increase of efflux on NCX, primarily due to that activated by Ca^2+^ waves. As shown in Fig. 5, in 1.8 mM Ca^2+^, the NCX efflux balances influx on the L‐type current. In contrast, in 10 mM Ca^2+^, efflux is 3.9 times influx though the L‐type current. We conclude, therefore, that the increase of SR Ca^2+^ content cannot be due to either an increase of Ca^2+^ entry on the L‐type current nor a decrease of efflux on NCX.

#### Background Ca^2+^ entry

Given that the cell must be in a steady state, there must be an additional component of Ca^2+^ influx to balance the increased efflux. We have estimated this background influx from the difference between measured influx and efflux in the steady state. Two points about this calculation needs addressing. (i) As in previous work, our electrophysiological approach measures Ca^2+^ efflux on the electrogenic NCX but not that on the electroneutral PMCA. It is likely that 10 mM external Ca^2+^ inhibits PMCA (Bassani *et al*. 1995) and we have therefore not corrected for this flux. (ii) The measurements of NCX current are made with respect to the baseline current and therefore ignore any contribution of NCX to this baseline. Both factors mean that our estimation of the background Ca^2+^ influx is, if anything, an underestimate. The data show that, not only is the background influx increased by elevation of external Ca^2+^ concentration, but (Fig. 5*B*), on average, it is greater in heart failure than control cells. More strikingly, the magnitude of this background influx determines whether or not waves occur. As shown in Fig. 5*C*, in control cells, those that show Ca^2+^ waves have a higher background influx than those that do not. This larger background influx in cells with Ca^2+^ waves is also apparent in heart failure cells. In summary, the magnitude of the background influx appears to be the single factor that is most correlated with whether or not waves occur and accounts for the bulk of the difference between control and heart failure.

Previous work on ventricular myocytes has shown that the refilling of an empty SR requires Ca^2+^ influx from outside the cell and a component of this occurs by a mechanism which does not involve either the L‐type Ca^2+^ current or NCX (Terracciano & MacLeod, 1996). A background Ca^2+^ entry pathway which is increased by hyperpolarization and is sensitive to Gd^3+^ has been identified (Kupittayanant *et al*. 2006). The fact that Ca^2+^ waves can be produced even when the cell membrane potential is held constant and there is no Ca^2+^ entry through the L‐type channel (Díaz *et al*. 1997) also argues for substantial background influx. In keeping with this previous work, our findings in spontaneously waving cells treated with nicardipine showed no role for Ca^2+^ entry via *I*_Ca‐L_ in generating waves, while waves were reduced by Gd^3+^. The effect of BI 749327 in both suppressing waves and reducing the background influx in Mn^2+^ quench experiments is strongly suggestive of a role for TRPC6 in mediating this background influx. In contrast, there was no evidence for other candidates such as connexin hemichannels or TRPC1/4/5 contrasting with the role for TRPC1/4 in promoting background Ca^2+^ entry in mouse ventricle (Camacho Londono *et al*. 2015).

### Study limitations

It has to be noted that, with the exception of Mn^2+^‐quench experiments, the background Ca^2+^ entry was studied under conditions of elevated Ca^2+^. While relevant to much other experimental work, it is unclear to what extent this background entry contributes under normal conditions. A major limitation to our understanding thus far has been the lack of knowledge of the identity of this background Ca^2+^ entry. As reviewed recently (Eisner *et al*. 2020), several candidates were proposed including Ca^2+^ entry through TRP channels (Camacho Londono *et al*. 2015) or connexin hemichannels (Wang *et al*. 2012). Our use of specific inhibitors has found a role for TRPC6 channels. We have not, however, examined other members of the TRP family. It is noteworthy that TRPC channel expression increases in heart failure (Bush *et al*. 2006; Kuwahara *et al*. 2006; Morine *et al*. 2016), and future work should address the role of specific mechanisms of these channels in generating the background influx under physiological conditions, as well as their contribution to adverse cardiac remodelling (Gao *et al*. 2012; Camacho Londono *et al*. 2015).

## Additional information

### Competing interests

No competing interests declared.

### Author contributions

Cellular experiments (D.C.H., B.C.N.); animal model (G.W.P.M., D.C.H., C.A.W., L.S.W.); experimental concepts, direction (D.C.H., D.A.E., A.W.T., K.M.D., E.F.B.); manuscript preparation (D.A.E., D.C.H., A.W.T.); funding (A.W.T., K.M.D., D.A.E.). All authors approved the final version of the manuscript. All authors revised the manuscript critically for important intellectual content. All experiments were performed at The University of Manchester. All persons designated as authors qualify for authorship, and all those who qualify for authorship are listed.

### Funding

The work was supported by grants from the British Heart Foundation: FS/15/28/31476, FS/12/57/29717, FS/09/036/27823, FS/20/6/34990, CH/2000004/12801, AA/18/4/34221 and Medical Research Council: MR/K501211/1. D.C.H. was supported by a clinical lectureship from the NIHR.

## Supporting information


Statistical Summary Document

Peer Review History

## Data Availability

The data that support the findings of this study are available from the corresponding author upon reasonable request.
